# Haemangiomas of the Small Intestine: Poorly Known Cause of Gastrointestinal Bleeding of Uncertain Origin

**DOI:** 10.7759/cureus.3155

**Published:** 2018-08-17

**Authors:** Wilmar A Ocampo Toro, Begoña Corral Ramos, Paula Concejo Iglesias, Jimena Cubero Carralero, David F Blanco García, Paola Barón Ródiz

**Affiliations:** 1 Radiology, Hospital Universitario Severo Ochoa, Leganés, ESP; 2 Radiology, Hospital Universitario Severo Ochoa, Madrid, ESP; 3 Radiology, Severo Ochoa University Hospital, Leganés, ESP

**Keywords:** cavernous haemangioma, benign tumour, small intestine, computed tomography, phleboliths, gastrointestinal bleeding

## Abstract

Small bowel haemangiomas are benign vascular tumours that may cause gastrointestinal bleeding of uncertain origin, are frequently chronic, and are rarely acute. We report a case of an haemangioma located in the distal ileum of a 29-year-old male with a history of chronic anemia since childhood. Imaging studies showed a mural thickening in the distal ileum with phleboliths, which is a key finding of haemangioma. Surgery was performed, and histology confirmed the diagnosis.

## Introduction

Haemangiomas are defined as vascular tumours that can appear anywhere on the body. In the small intestine they account for 7-10% of haemangiomas of the gastrointestinal tract and may be a cause of bleeding of uncertain origin (defined as chronic or recurrent gastrointestinal bleeding of unknown cause) or cause massive bleeding [1-4].

Its location in the small intestine explains why the diagnosis of these tumours has traditionally been possible only after surgery. Since the development of computed tomography (CT) and new balloon-guided endoscopic techniques, preoperative diagnosis has become more frequent.

## Case presentation

**Figure 1:**
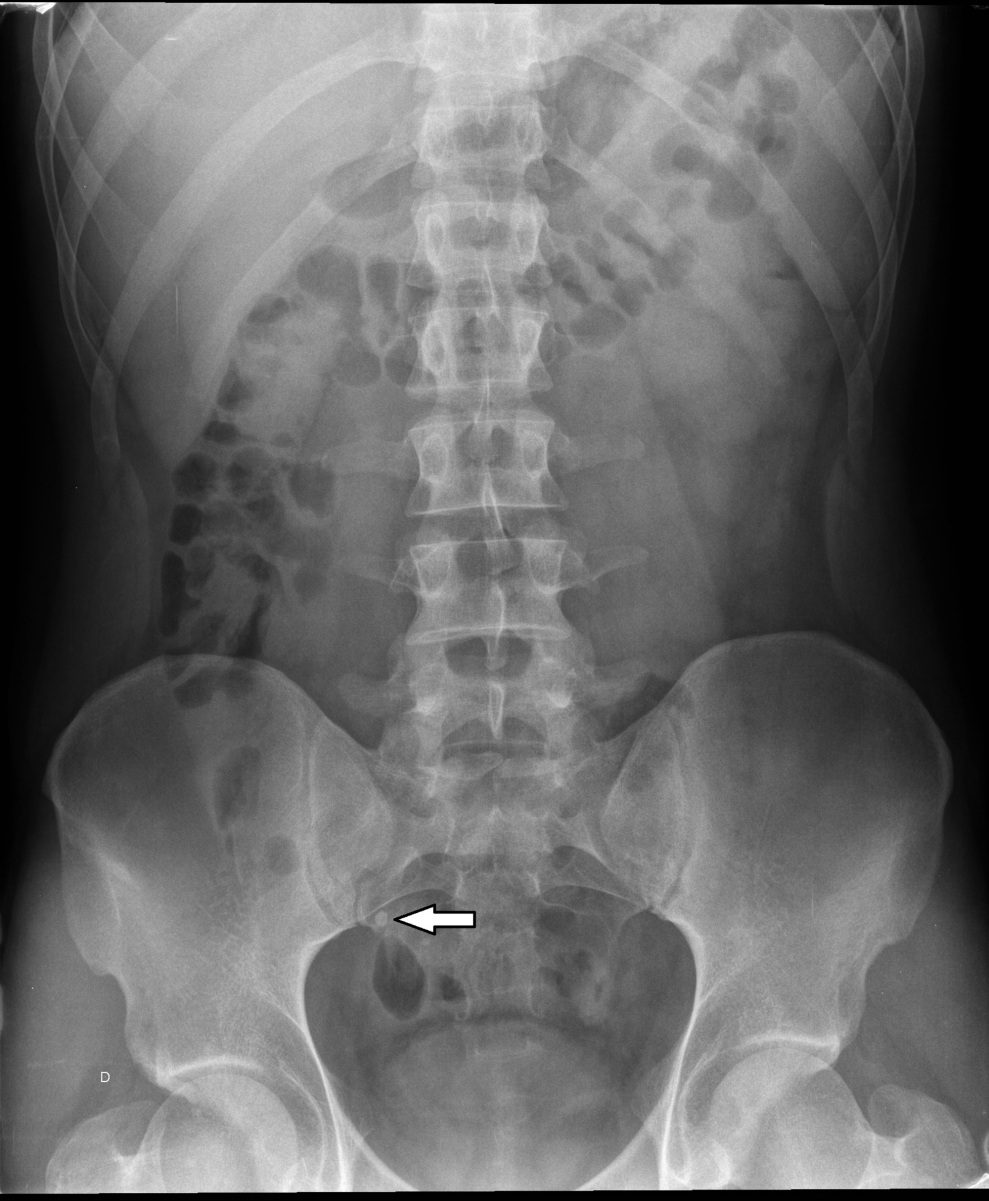
Simple abdominal X-ray. Phebolites are shown overlying the right sacrum (white arrow).

A 29-year-old male presented at our hospital with a history of chronic anemia since childhood. The patient underwent gastroscopy and colonoscopy with normal results. Due to the persistence of the anemia, despite symptomatic treatment with oral iron, it was decided to extend the study with imaging techniques. A simple abdominal X-ray was performed, and although at first it did not reveal any significant alterations, retrospectively, phleboliths were visualised in the right side of the pelvis (Figure 1).

**Figure 2:**
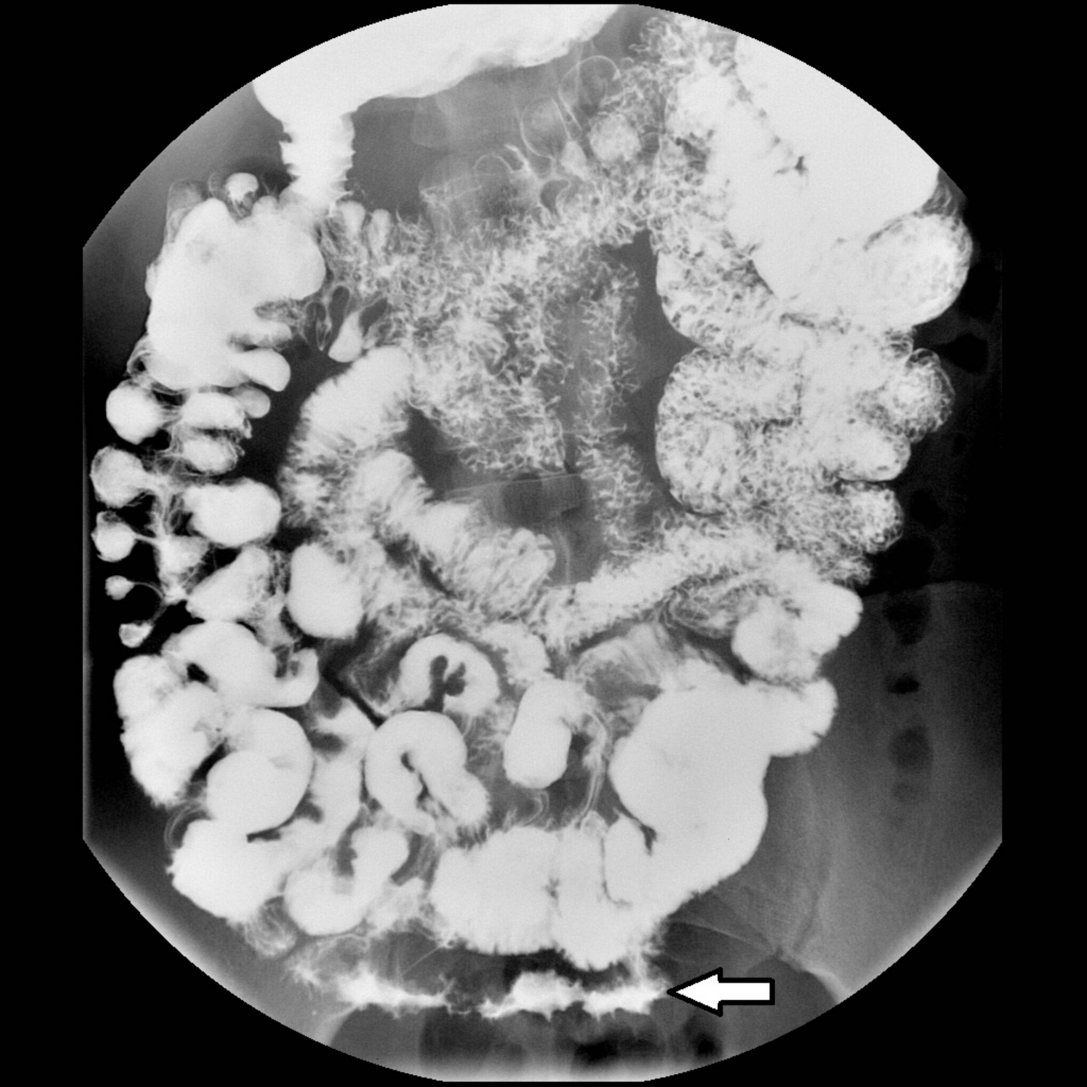
Small bowel study with barium. Irregular contour thickening is shown in the ileum (white arrow).

A small bowel study (SBS) with barium was also performed, and it showed in the distal ileum the poor distensibility and irregular contour (Figure 2).

**Figure 3:**
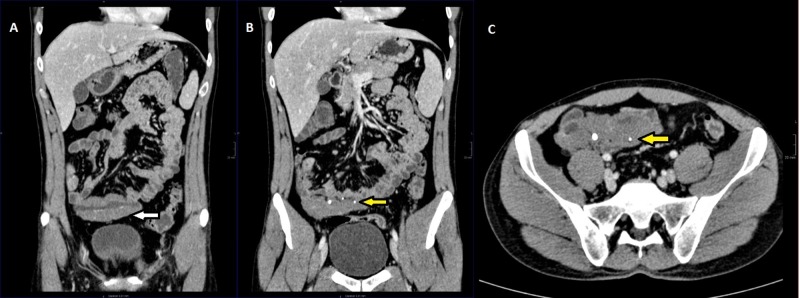
Two coronal and one axial reconstructions of computed tomography enterography. A, Coronal reconstruction shows the ileum mural thickening (white arrow). B, Coronal reconstruction depicts phleboliths (yellow arrow) and the ileum mural thickening. C, Same findings are showed on the axial reconstruction.

Because of these findings, computed tomography enterography (CTE) was performed, which showed phleboliths and transmural wall thickening in a 10-cm segment of the distal ileum with homogeneous contrast enhancement (Figure 3).

No inflammatory changes were seen in mesenteric fat. No abdominal lymph nodes suggestive of malignancy were seen, and there was no involvement of adjacent organs.

Based on these findings, the most likely preoperative diagnosis was haemangioma of the distal ileum. A laparotomy and segmental small bowel resection was performed, which revealed a 10-cm ileum lesion that on pathology examination suggested a vascular nature due to its bluish purple coloration, compressibility, and presence of varices on its surface. The histopathological assessment revealed a vascular lesion compatible with haemangioma. After our patient recovered from surgery, he remained asymptomatic.

## Discussion

Haemangiomas are hamartomatous vascular benign tumours that can appear anywhere in the gastrointestinal tract [1, 5, 6]. Although their occurrence in the gastrointestinal tract is rare, the small intestine is the most frequent site, and it accounts for 7-10% of benign neoplasms in this location and 0.05% of all gastrointestinal tract neoplasms [1, 2, 4, 5]. They are more frequent in young patients (5-25 years) [1] with a male:female ratio of 1:2.5 and possible spontaneous involution during childhood [6, 7]. They can be single or multiple [1, 4, 6-8].

Multiple haemangiomas are usually associated with cutaneous haemangiomas and syndromes such as blue rubber syndrome, Osler-Weber-Rendu syndrome, Maffucci syndrome, Klippel-Trenaunay-Weber, among other syndromes [4, 7]. These syndromes may be associated with haemangiomatosis, which consists of the presence of multiple haemangiomas that infiltrate the wall of the gastrointestinal tract, the mesentery, and sometimes even the retroperitoneum, also involving solid abdominal viscera [4].

At pathologic assessment, haemangiomas generally involve the intestinal wall from the submucosa to the muscular layer [1, 4], although they sometimes extend beyond the serosa into the mesenteric, retroperitoneal, or pelvic fat [4, 9]. They may rarely present as bluish-reddish submucosal nodules or polyps, which may be located either in a variable length segment of a loop or distributed throughout the gastrointestinal tract [1, 4]. Microscopically, most haemangiomas originate from submucosal vascular plexus [7]. There are three histological types: cavernous (the most frequent type), capillary, or mixed [1, 4, 8, 9]. Capillary haemangiomas are a proliferation of small capillaries composed of thin-walled vessels lined by endothelial cells, while cavernous haemangiomas consist of large sinuses lined by multiple layers of endothelial cells. Mixed haemangiomas are a mixture of both [6]. There seems to be a correlation between the type of haemangioma and the type of bleeding. The capillary type is more frequently associated with chronic bleeding, while the cavernous one is associated with massive bleeding [3].

Clinically, haemangiomas are symptomatic in 90% of the cases, unlike other benign tumours of the small intestine that tend to present as an incidental finding [10]. The most frequent sign is chronic gastrointestinal bleeding, causing anemia of unknown origin and rarely massive bleeding [1, 2, 4, 6-9].

Occasionally, these may cause intestinal obstruction, invagination, or recurrent abdominal pain and rarely platelet sequestration [8]. Some of the complications are perforation and malabsorption syndrome.

The diagnosis of these lesions is complex and there is no image study, to date, which allows us to reach the definitive diagnosis. Since the most frequent clinical presentation in these patients is gastrointestinal bleeding, they frequently undergo gastroscopy and colonoscopy studies with normal results as in the exposed case [8]. A simple abdominal X-ray may be useful, if phleboliths (50% of cases), obstruction, or perforation is present [1, 4].

In our case, phleboliths were visible in both simple abdominal X-ray and CTE. Phleboliths are secondary to thrombosis of the intralesional vessels and subsequent partial or total calcification of thrombus. Phleboliths are virtually pathognomonic of haemangioma if they are grouped and when they are seen in young patients, becoming key in the diagnosis of such cases [4, 6-8]. SBS are useful to visualize submucosal lesions, showing an image of defective filling of nodular or polypoid morphology or even luminal narrowing by diffuse involvement of the wall in a segment of small intestine as in our case (Figure 2) [8]. In addition, air insufflation into the intestinal lumen demonstrates compressibility of the lesion, which is highly suggestive of vascular lesion [1, 4, 6]. However, some thrombosed haemangiomas or lesions with phleboliths are poorly compressible [4]. In cases of larger haemangiomas, we can see the displacement of the intestinal loops [8].

In non-intravenous (IV) contrast-enhanced computed tomography, haemangiomas may have a nodular or polypoid appearance or appear as wall thickening. Lesions show heterogeneous density (due to the existence of thrombosed zones) usually with luminal growth and rarely extending to the adjacent tissues. The non-IV contrast-enhanced CT can show the presence of phleboliths, which can be seen better than after the administration of intravenous contrast [7].

Computed tomography with intravenous contrast (contrast-enhanced computed tomography/CECT) is a fundamental tool, especially in emergency situations, because of its speed, availability, and its ability to diagnose extra-intestinal pathologies [1]. In the arterial phase, the lesions are hypervascular with a peripheral, progressive, and discontinuous contrast uptake, like hepatic haemangiomas and haemangiomas in other locations [7, 10]. Portal phase uptake tends to be homogeneous or central. In cases of infiltration of the mesenteric fat, areas of increased density can be seen, corresponding to affected areas by the tumour. CECT also reveals engorgement of the mesenteric vessels adjacent to the lesion [7].

CTE allows a better evaluation of the intestinal lumen by using negative luminal contrast (usually water associated with an osmotically active component: polyethylene glycol or methylcellulose), which allows the distension of the loops, improving the detection and characterisation of diseases in the small intestine [7].

To evaluate the colon and upper gastrointestinal tract, endoscopy and colonoscopy studies are used. Some lesions in the duodenum and ileum may be accessible. These techniques reveal bluish or reddish submucous that are compressible with air insufflation [1, 2, 6]. The presence of varices and telangiectasias suggests the diagnosis of haemangioma. The suspicious diagnostic is modified according to the experience of the endoscopist and, in some cases, biopsy can be avoided (which has a considerable risk of bleeding) [4, 6].

Endoscopic ultrasound exposes haemangiomas in the duodenum, allowing to define its submucosal origin and its degree of local extension to adjacent organs [7].

Magnetic resonance imaging (MRI) shows wall thickening with low signal intensity on T1-weighted images and heterogeneous bright signal intensity on T2-weighted images. This last finding is more evident in fat suppression sequences [1, 6]. These lesions do not restrict in diffusion [11]. Phleboliths are usually signal void on T1 and T2 weighted images. However, CT has more sensibility in its detection [1].

MRI unlike CT can demonstrate blood flow in the lesion without medium contrast administration [1]. The endoscopic capsule may be useful in the study of patients with recurrent gastrointestinal bleeding [1, 3, 9]. However, 30% of the results it produces are false positives and 20% of the examinations are incomplete. In addition, retention of the capsule in the intestine may be possible [10].

The differential diagnosis of haemangioma includes both benign and malignant tumours: lipoma, leiomyoma, lymphangiomas or adenomas as benign tumours and adenocarcinoma, gastrointestinal stromal tumours, lymphoma, carcinoid tumours or metastases as malignant lesions (more frequent than benign tumours) [1]. Other causes of gastrointestinal bleeding of obscure origin such as Meckel diverticulum or other causes of mesenteric and small bowel infiltration—malignant mesentery tumours, fibromatosis, and inflammatory pseudotumor—should be also ruled out [4].

Biopsy of small bowel haemangiomas has a very limited role due to the difficult access of these tumors and the high risk of uncontrollable bleeding [3, 4, 11].

The treatment of choice of haemangiomas is surgical and resection of the affected segment [1, 3, 4, 6]. In our case laparotomy and resection of the affected segment of the ileum were performed. In general, CTE is the best imaging method for preoperative localization of small bowel haemangiomas.

In some cases of polypoid lesions accessible to endoscopy, it may be possible to perform polypectomy and cauterisation [1, 2, 9]. However, it is a controversial option because of the risk of uncontrollable bleeding and intestinal perforation. In some cases of non-controllable haemorrhage, haemangioma can be embolised. Some haemangiomas may involute during childhood or if they are treated with corticosteroid or gamma interferon [6]. There is no evidence in the literature of the recurrence of haemangioma [5, 9].

## Conclusions

Small bowel haemangiomas are a rare cause of gastrointestinal bleeding. Diagnosis is often delayed or incorrect; so it is important that these tumours are considered in the differential diagnosis during the study of an anemia of uncertain origin.

Diagnosis of these tumours is difficult at the beginning and a wide range of diagnostic studies (mainly radiological and endoscopic) are needed and performed according to the features of the patient and the lesion. CTE is becoming the most accurate diagnostic technique for these tumours. Definitive diagnosis is histological and the treatment of choice is surgery.

## References

[1] Corsi A, Ingegnoli A, Abelli P (2007). Imaging of a small bowel cavernous hemangioma: report of a case with emphasis on the use of computed tomography and enteroclysis. Acta Biomed Ateneo Parmense.

[2] Attash SM, Ali MS, Al-Nuaimy HA (2012). Isolated cavernous haemangioma of the stomach in a 3-year-old child: an unusual cause of upper GI bleeding. BMJ Case Reports.

[3] Yoo S (2011). GI-associated hemangiomas and vascular malformations. Clin Colon Rectal Surg.

[4] Chen HH, Tu CH, Lee PC, Chiu HH, Wu MS, Wang HP (2015). Endoscopically diagnosed cavernous hemangioma in the deep small intestine: a case report. Advances in Digestive Medicine.

[5] Fernandes D, Dionísio I, Neves S, Duarte P (2014). Cavernous hemangioma of small bowel: a rare cause of digestive hemorrhage. Rev Esp Enferm Dig.

[6] Levy AD, Abbott RM, Rohrmann JC A, Frazier AA, Kende A (2001). Gastrointestinal hemangiomas: imaging findings with pathologic correlation in pediatric and adult patients. AJR.

[7] Lee NK, Kim S, Kim GH, Jeon TY, Kim DH, Jang HJ, Park DY (2010). Hypervascular subepithelial gastrointestinal masses: CT-pathologic correlation. Radiographics.

[8] Varma JD, Hill MC, Harvey LA (1998). Hemangioma of the small intestine manifesting as gastrointestinal bleeding. Radiographics.

[9] Peng C, Chen H, Li W, Xu R, Zhuang W (2016). A rare cause of recurrent gastrointestinal bleeding: giant diffuse and cavernous intestinal mesentery hemangioma in an adult. Dig Dis Sci.

[10] Huprich JE, Barlow JM, Hansel SL, Alexander JA, Fidler JL (2013). Multiphase CT enterography evaluation of small-bowel vascular lesions. AJR.

[11] Si-Mohamed S, Aufort S, Khellaf L, Ramos J, Gasne P, Durand L (2015). Mesenteric cavernous hemangioma: imaging-pathologic correlation. Diagn Interv Radiol.

